# Targeting Cutaneous Leishmaniasis with Thiadiazine Thione Derivatives: An In Vivo Study of Its Anti-Inflammatory, Anti-Pyretic, Anti-Nociceptive, and Anti-Sedative Properties

**DOI:** 10.3390/biomedicines13010093

**Published:** 2025-01-03

**Authors:** Sarah Sarwar, Nadia Sarwar, Haleema Ali, Rafiullah, Rasool Khan, Ajaz Ahmad, Amin Ullah, Ho Soonmin, Nazif Ullah

**Affiliations:** 1 Department of Biotechnology, Abdul Wali Khan University Mardan, Mardan 23200, Pakistan; sarasarwar86@gmail.com; 2 Department Physiology and Medical Physics, Royal College of Surgeons, D02 YN77 Dublin, Ireland; nadiasarwar@rcsi.com; 3 Institute of Chemical Sciences, University of Peshawar, Peshawar 25120, Pakistan; haleema.chemist@gmail.com (H.A.); rasoolkhan@uop.edu.pk (R.K.); 4 Principal Research Officer, Veterinary Research Institute, Peshawar 25000, Pakistan; drrafi_ullah@yahoo.com; 5 Department of Clinical Pharmacy, College of Pharmacy, King Saud University, Riyad 11451, Saudi Arabia; ajukash@gmail.com; 6 Department of Allied Health Sciences, Iqra National University, Peshawar 25100, Pakistan; aminbiotech7@gmail.com; 7 Faculty of Health and Life Sciences, INTI International University, Putra Nilai 71800, Malaysia; soonmin.ho@newinti.edu.my

**Keywords:** anti-inflammatory, good health, *Leishmania tropica*, thiadiazine thione derivatives, toxicological study, in vivo activity

## Abstract

**Background/Objectives:** Thiadiazine thione (THTT) has gained significant interest owing to its pharmacological potentials, particularly its antiparasitic and anti-inflammatory properties. Leishmaniasis is a clinical syndrome caused by infection with *Leishmania* species and is associated with an inflammatory response and nociception. The available treatments against leishmaniasis are inadequate, as they are associated with high cost, toxicity, and increased resistance. **Methods:** In the current study, the antileishmanial potential of five Thiadiazine thione derivatives (C1–C5) was evaluated in vivo against *Leishmania tropica*. Experiments were performed on BALB/c mice infected with promastigotes and treated with THTT derivatives for 15 days. Additionally, the derivatives were evaluated for their anti-inflammatory, antinociceptive, antipyretic, and antisedative properties using standardized models, including carrageenan-induced paw edema, acetic acid-induced abdominal writhes, yeast-induced fever, and white wood apparatus, respectively. **Results:** Of the tested derivatives, C5 exhibited the most promising results, with a 61.78% reduction in lesion size and significant decrease in parasite load. Among the derivatives, C1 showed the highest anti-inflammatory activity, with 63.66% inhibition in the paw edema test at the 5th hour post treatment. In the antipyretic assay, C1 and C5 were able to reduce body temperature to a normal level within 1 h of treatment. Furthermore, compounds C4, C2, and C1 showed high nociceptive activity, while C1 and C5 demonstrated the most notable antisedative effects (94 ± 2 and 92 ± 1, respectively), outperforming the standard drug diazepam (13 ± 1). **Conclusion:** These in vivo findings suggest that THTT derivatives have the potential to serve as a template for developing leishmanicidal drugs, with added anti-inflammatory and analgesic properties.

## 1. Introduction

Parasitic diseases affect hundreds of millions of people worldwide and remain a major public health concern [1]. These diseases includes trypanosomiasis, pediculosis, trichomoniasis, malaria, giardiasis, and leishmaniasis, all caused by different Protozoans [2]. Leishmaniasis, a disease caused by infection with *Leishmania* species, is particularly prevalent in developing countries of tropical and subtropical regions, where it is transmitted by the bite of infected sandflies, transmitting a unicellular eukaryotic flagellated parasite to humans through the bite, which is then converted to a proliferative amastigote form inside the host cells [3]. Clinical manifestation varies depending upon the type of infection and *Leishmania* species, for example, cutaneous, mucocutaneous, and visceral leishmaniasis [3]. The most common form is cutaneous leishmaniasis (CL). Leishmanial infections are considered endemic in more than ninety countries of the world [4]. Cutaneous leishmaniasis causes skin lesions, mainly ulcers on the body surfaces, with lifelong scars and serious disabilities, whereas in mucocutaneous leishmaniasis (MCL), complete or partial destruction of the mucus membrane of the mouth, nose, and throat occurs [5]. Cutaneous leishmaniasis is caused by distinct parasite species, such as *L. major*, *L. tropica*, *L. braziliensis*, *L. aethiopica*, and *L. mexicana*, among others, while VL is caused by *L. donovani* and *L. infantum* species [6]. The diagnosis of leishmaniasis is generally based on clinical signs and symptoms, microscopic detection of the parasites, and tissue culture. The reported causative agent of anthroponotic CL in Pakistan is mainly *Leishmania tropica* (*L. tropica*), which is seen in urban areas, whereas the agent for zoonotic CL is *Leishmania major* (*L. major*), which is more common in rural areas [7]. Prevalence of leishmaniasis is also high in other developing countries of Africa, Asia, and Latin America [8]. Cutaneous leishmaniasis is considered a major public health concern in Pakistan. Over 90% of CL cases were reported in Pakistan, Iran, and Afghanistan [7]. The high cost of available treatments, limited health facilities, poverty, and increasing drug resistance complicate efforts to control parasitic infections in these areas [9].

Despite significant advances in exploring the biology, etiology, and pathophysiology of *Leishmania*, no vaccine is currently available to prevent leishmaniasis [10]. Transmission is challenging to control due to the zoonotic nature of the infection and the difficulty in reducing sandfly populations, although some attempts have been made to reduce the vector (sandfly) [11]. Existing chemotherapy options, such as pentavalent antimonials, amphotericin B, and pentamidine, are inadequate due to their limited efficacy and toxicity and the growing resistance of *Leishmania* parasites. Furthermore, varying strains of *Leishmania* exhibit different levels of sensitivity to existing drugs, emphasizing the critical need for discovering new, effective treatments [12]. *Leishmania tropica* and *Leishmania major* are the significant targets for drug discovery due to their high prevalence in Pakistan. Anthroponotic transmission and association with disfiguring cutaneous lesions make it a pressing public health concern in the region. However, *Leishmania tropica* was targeted in the current study due to the availability of its well-characterized strain.

Inflammation plays a crucial role in the disfiguring and mortality of patients with various forms of leishmaniasis. Upon infection, *Leishmania* parasites invade macrophages and other immune cells, triggering a pro-inflammatory immune response characterized by the release of cytokines and chemokines. This results in tissue damage, ulcer formation, and exacerbated inflammation [13]. Therefore, controlling inflammation and managing the immune response are essential for preventing mortality and improving the overall prognosis of patients with leishmaniasis [14]. Therefore, a series of pharmacological activities was also carried out in the present study.

A new approach in search of antileishmanial drugs is the design of prodrugs that could inhibit *Leishmania*-specific critical enzymes. Heterocyclic compounds are well known for their antiparasitic potential. Nitrogen-containing heterocyclic compounds are commonly found in many commercially available natural and synthetic drugs [15,16]. To combat emerging drug resistance, it is critical to develop new derivatives through the structural diversification of existing compounds. Thiadiazine thione (THTT) has gained significant interest owing to its pharmacological potentials, particularly its antiparasitic and anti-inflammatory properties. The introduction of various substituents to the thiadiazine thione scaffold may improve the potency and decrease toxicity. Thiadiazine thione (THTT) derivatives have been reported for their promising medicinal properties, which include antibacterial, antifungal, antihelmintic, antiprotozoal, tuberculostatic, herbicidal, and antioxidant activities [17,18]. In addition to antimicrobial activity, these compounds have a place in the treatment of arteriosclerosis and possess antifibrinolytic, cytotoxic, and antiepileptic activity [19,20,21]. Thiadiazine thione derivatives have also been studied as potential components of prodrugs for different biological activities. Thus, antibiotic drugs like ampicillin, amoxicillin, and cephalexin have been incorporated within the thiadiazine thione nucleus to create prodrugs [22].

Taken together the pharmacological importance of THTTs and higher prevalence of cutaneous leishmaniasis, the current study focused on investigating the leishmanicidal activity against *Leishmania tropica* (due the availability of a well-characterized strain) and the anti-inflammatory, antipyretic, anti-analgesic, and antisedative potential of our THTT derivatives in BALB/c mice in an in vivo experiment.

## 2. Materials and Methods

### 2.1. Drugs

**Figure 1:**
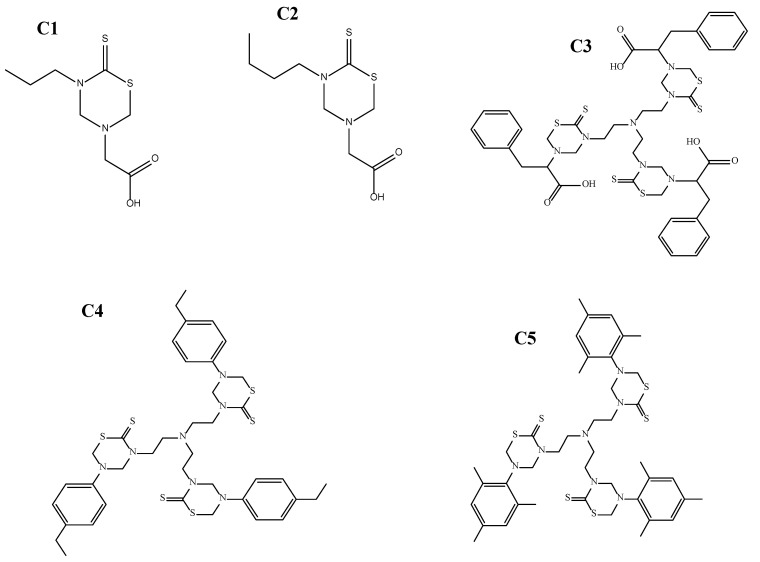
The 2-(5-propyl-6-thioxo-1,3,5-thiadiazinan-3-yl)acetic acid (**C1**), 2-(5-Butyl-6-thioxo-1,3,5-thiadiazinan-3-yl) acetic acid (**C2**), 2,2′,2″-(5,5′,5″-(nitrilotris(ethane-2,1-diyl))tris(6-thioxo-1,3,5 thiadiazinane-5,3-diyl))tris(3-phenylpropanoic acid) (**C3**), 5,5′,5″-(nitrilotris(ethane-2,1-diyl))tris(3-(4-ethylphenyl)-1,3,5-thiadiazinane-2-thione) (**C4**), and 3,3′,3″-(nitrilotris(ethane-2,1-diyl))tris(5-mesityl-1,3,5-thiadiazinane-2-thione) (**C5**).

The Thiadiazine thione derivatives used in the current study were provided by the Institute of Chemical Sciences (ICS), University of Peshawar, Pakistan. In total, five compounds were selected based on their in vitro antileishmanial activity against promastigote form of *Leishmania tropica*. Two of the derivatives, C1 and C2, are mono-THTT derivatives reported against *Leishmania major* [23], while the three compounds C3, C4, and C5 are tris-THTT derivatives, which are novel compounds and reported in the current study for the first time. The *log* p-values for C1 and C2 are <5, while those for C3, C4, and C5 are slightly greater than 5. The IUPAC names are given below:

2-(5-propyl-6-thioxo-1,3,5-thiadiazinan-3-yl)acetic acid (C1),
2-(5-Butyl-6-thioxo-1,3,5-thiadiazinan-3-yl) acetic acid (C2),
2,2′,2″-(5,5′,5″-(nitrilotris(ethane-2,1-diyl))tris(6-thioxo-1,3,5 thiadiazinane-5,3-diyl)) tris(3-phenylpropanoic acid) (C3), 5,5′,5″-(nitrilotris(ethane-2,1-diyl))tris(3-(4-ethylphenyl)-1,3,5 -thiadiazinane-2-thione) (C4), and 3,3′,3″-(nitrilotris(ethane-2,1-diyl))tris(5-mesityl-1,3,5-thiadiazinane-2-thione) (C5) (Figure 1).

### 2.2. Animals

In vivo assays were conducted on female BALB/c mice (4–6 weeks old; 18–20 g) provided by the Veterinary Research Institute (VRI), Peshawar, Khyber Pakhtunkhwa, Pakistan. Animals were housed under standard conditions (25–26 °C) at the animal facility of VRI, with a 12 h light/dark cycle and free access to food and water.

All experimental procedures and protocols were approved by the Ethics Committee for Animal Research of Abdul Wali Khan University, Mardan, Pakistan, AWKUM/BIOTECH/2022/2930, 11 May 2022. Mice were maintained in polypropylene cages with free access to food and water, 50–70% relative humidity, and a 12 h light/dark cycle (light from 9 a.m.). Experiments were carried out between 10 a.m. and 6 p.m. Animals were segregated into 17 groups, and for each group, the number of mice was (*n* = 6). The groups included the following: control group receiving Phosphate buffered saline (PBS) or Normal saline (*n* = 6); standard drug-receiving group (*n* = 6); group receiving 100 mg/kg body-weight dose of test compounds (C1, C2, C3, C4, and C5) (*n* = 6 × 5); group receiving 50 mg/kg body-weight dose of test compounds (C1, C2, C3, C4, and C5) (*n* = 6 × 5); and group receiving 25 mg/kg body-weight dose of test compounds (C1, C2, C3, C4, and C5) (*n* = 6 × 5). Animals were euthanized one (*n* = 6 per group) and fifteen (*n* = 6 per group) days after treatment, when biochemical, histological, and parasitological analyses were performed.

#### Inclusion and Exclusion Criteria

Mice of same age and weight were used to ensure uniformity in all experiments. In the current study, only female (BALB/c) mice were used to minimize the genetic and hormonal variations. All the animals with pre-existing conditions, such as tumors or wounds, were excluded. Mice that lost or gained 10% of their initial body weight or that showed any other behavioral abnormalities, such as lethargy, loss of appetite, etc., were excluded from the study.

### 2.3. Parasite Culture

The *Leishmania tropica* strain (KWH23) was provided by the Department of Biotechnology, Abdul Wali Khan University, Mardan. Parasites were cultivated in RPMI-1640 medium enriched with 20% (*v*/*v*) heat-inactivated fetal bovine serum (FBS; Sigma-Alderic, St. Louis, MO, USA) and a 1% solution of penicillin and streptomycin at 25 ± 1 °C, pH 7.4 [24].

### 2.4. Acute Toxicity Test

An acute toxicity test was conducted to determine the safe dose range for the THTT derivatives. Female BALB/c mice were injected intraperitoneally with doses ranging from 25 to 1000 mg/kg (*n* = 6 for each dose). Mice were monitored for 24 h for behavioral changes like aggressiveness, writhing, convulsions, or ataxia [25,26].

### 2.5. Therapeutic Dose Selection

Therapeutic doses were selected based on a previously reported method [27,28]. The lowest effective dose was determined to be 25 mg/kg, where significant therapeutic effects were observed.

### 2.6. In Vivo Antileishmanial Assay

The in vivo antileishmanial assay was performed as described by Ghasemi et al., 2019 [29]. Mice were infected subcutaneously with stationary-phase promastigotes (1 × 10^6^ parasites). When nodules developed at the injection site (40–50 days post infection), animals were grouped as given below:

Group I received 200 µL PBS (i.p route) daily for 15 days (control; *n* = 6); Group II received 50 µL standard drug Amp B 20 mg/kg daily (IM route) (*n* = 6); Group III received 50 µL test compounds at a concentration of 100 mg/kg (*n* = 6 × 5); Group IV received 50 mg/kg (*n* = 6 × 5); and Group V received 25 mg/kg (*n* = 6 × 5). These regimens of all five derivatives (C1–C5) were administered subcutaneously every two days for a period of 10 days.

From each group, four mice were selected to measure the lesion size with confirmed infection status. To check the drug efficacy, the width and length of the lesion were measured weekly by vernier caliper. The size (mm) was determined in two diameters (D and d) at a right angle to each other according to the formula S = (D + d)/2 [30]

#### 2.6.1. Parasite Burden by Microscopic Examination

Parasite load in the lesions was assessed using the method outlined by Ezatpour et al., 2015 [31]. After cleaning the lesions with ethanol, the margins were punctured with a sterile lancet, and the exudation material was collected for smearing. The smears were air-dried, fixed with methanol, and stained with Giemsa to determine the parasite load under light microscopy.

Negative: 0 parasite/10 fields.

1+: 1 parasite/10 fields.

2+: 1–10 parasites/10 fields.

3+: 10–100 parasites/10 fields.

4+: 101–1000 parasites/10 fields.

5+: >1000 parasites/10 fields.

#### 2.6.2. Toxicological Study

The toxicity of all compounds was evaluated in vivo in sera samples obtained from infected and treated mice one (*n* = 6/group) and fifteen days post treatment. Liver function was analyzed by enzymes aspartate aminotransferase (AST) and alanine aminotransferase (ALT) as hepatic damage markers. Sera samples of non-treated and non-infected mice (*n* = 6) were used as control, while the levels of blood urea nitrogen and serum creatinine were evaluated to access the renal function as previously described by Mendonca et al., 2016 [32]. Evaluations were performed using commercial kits (Labtest Diagnostica^®^, Belo Horizonte, Brazil) according to the manufacturer’s instructions.

### 2.7. Histopathology

#### Dissection Procedure of Animals and Slides Preparation

Mice were kept on normal feed for at least 24 h before dissection and were euthanized 15 days after treatment under aseptic conditions. Blood was collected in EDTA tubes for serum preparation. Organs (liver and kidney) were removed for histopathological examination. Organs were washed with saline, blotted dry, and weighed. Tissue samples were fixed in 10% formalin for further processing [33]. Small tissue pieces were fixed for 3–4 h in fixative sera, followed by dehydration with alcohol and transfer to cedar wood oil. Tissues were embedded in paraplast, and 3–4 µm thin slides were prepared using a microtome. After removing the wax, tissues were stained with hematoxylin-eosin, and images were captured using a Nikon eclipse E200 microscope [34].

### 2.8. In Vivo Pharmacological Efficacy Assessment

#### 2.8.1. Carrageenan-Induced Inflammation

The anti-inflammatory potential of THTT derivatives was assessed by using the carrageenan-induced paw edema model in mice [35]. Mice were divided into the following groups:

Group I: Normal saline (0.9% NaCl) (*n* = 6).

Group II: Standard drug, aspirin (50 mg/kg in saline) (*n* = 6).

Group III: 50 µL test compounds at a concentration of (100 mg/kg) (*n* = 6 × 5); Group IV received 50 mg/kg (*n* = 6 × 5); Group V received 25 mg/kg (*n =* 6 × 5).

Mice were injected subcutaneously with 1% w/v carrageenan (0.05 mL) into the right hind paw, and the paw volume was measured at 1–5 h using a Plethysmometer (LE 7500 plan Lab S.L). The percentage inhibition of inflammation was calculated using the following formula:

$$Percentage\;inhibition=\left(C-\frac{T}{C}\right)\times 100$$

where *C* is the average inflammation of control, and *T* is the paw volume of the tested group.

#### 2.8.2. Antinociceptive Study

The analgesic potential of THTT derivatives was evaluated by acetic acid-induced writhing test [36]. Mice were randomly divided into the groups given below (*n* = 6). All treatments were given through i.p. route. After 30 min of the treatments, acetic acid (1%) was injected to all mice, and a few minutes later, abdominal writhing was noted for 10 min.

Group I: Saline (10 mL/kg) (*n* = 6).

Group II: Aspirin (standard drug, 50 mg/kg) (*n* = 6).

Group III received 50 µL test compounds at a concentration of (100 mg/kg) (*n* = 6 × 5); Group IV received 50 mg/kg (*n* = 6 × 5); Group V received 25 mg/kg (*n* = 6 × 5).

#### 2.8.3. Antipyretic Activity by Brewer’s Yeast Method

The antipyretic potential of THTT derivatives was evaluated using the Brewer’s yeast method **[37]**. Normal body temperatures of mice were recorded by inserting the digital thermometer 3–4 cm deep into rectum. Mice were injected (subcutaneously) with 15% w/v yeast suspension (10 mL/kg), and their rectal temperature was measured 19 h later. The animals were divided into the following groups:

Group I: Saline (5 mL/kg) (*n* = 6).

Group II: Aspirin (standard drug 100 mg/kg) (*n* = 6).

Group III received 50 µL test compounds at a concentration of (100 mg/kg) (*n* = 6 × 5); Group IV received 50 mg/kg (*n* = 6 × 5); Group V received 25 mg/kg (*n* = 6 × 5).

The rectal temperature was recorded at 1 h intervals for up to 5 h after treatment [38].

#### 2.8.4. Antisedative Effect

The antisedative effect of THTT derivatives was assessed by using the white wood apparatus, which is enclosed in stainless steel and divided by black lines. Mice were exposed to red light for one hour with feed supply. Mice were divided into the groups mentioned below. Treatments were given through i.p. route, and after 30 min, each mouse was positioned in the center of wooden box, and the number of lines crossed by each animal was recorded [39].

Group I: Saline (10 mL/kg) (*n* = 6).

Group II: Diazepam (standard drug 0.5 mg/kg) (*n* = 6).

Group III received 50 µL test compounds at a concentration of (100 mg/kg) (*n* = 6 × 5); Group IV received 50 mg/kg (*n* = 6 × 5); Group V received 25 mg/kg (*n* = 6 × 5).

##### Statistical Analysis

Statistical analysis was performed using GraphPad Prism 9, applying one-way ANOVA. The normality assumptions for the response variables were confirmed through Shapiro–Wilk normality [39,40,41]. Data are expressed as ± standard deviation (SD), and statistical significance was set at *p* < 0.05. All experiments were conducted in triplicates, and the graphs were constructed using the mean values, with error bars representing the standard deviation.

## 3. Results

### 3.1. Assessment of Safety Profile

During the initial acute toxicity evaluation, THTT derivatives (as shown in Figure 1) were found to be safe by monitoring the animal’s behavior for 2, 4, 6 h and then up to 24 h post treatment. No animals exhibited abnormal behavior, such as aggressiveness, writhing, convulsions, or ataxia, and no mortality was observed at doses up to 500 mg/kg. These findings indicate that all five compounds were safe up to 500 mg/kg body weight, which was considered the maximum tolerated dose (MTD).

### 3.2. In Vivo Antileishmanial Activity

#### Lesion Size and Parasite Load

**Figure 2:**
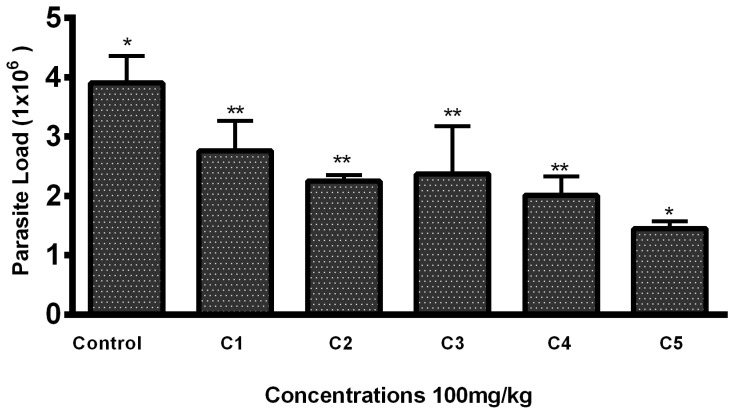
Parasite load in mice lesion sample after treatment with the THTT derivatives. Bars indicate the mean plus standard deviation of the groups. (*) and (**) indicate statistically significant difference in relation to the non-treated control * *p* < 0.01; ** *p* < 0.05.

**Figure 3:**
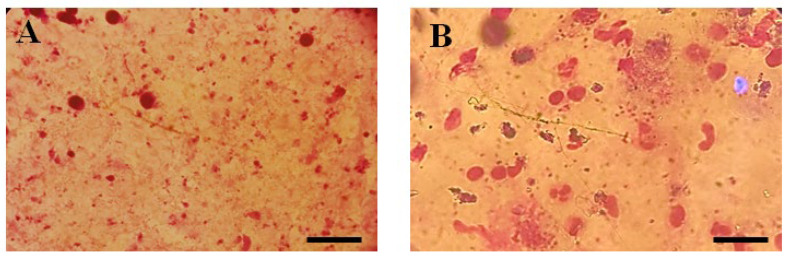
Microscopic illustration of Parasite Burden in Blood Samples Stained with Giemsa. (**A**) Un-treated and (**B**) Parasite load of Thiadiazine thione-treated Sample with 40X Magnification (Scale bar = 10 µm).

Lesions developed at the site of injection 45–50 days post infection. Mice with 1–1.5 cm lesion size were selected from each group and treated with THTT derivatives. Mice with severe lesions and or showing signs of secondary infections were excluded. Results indicated that the lesion size was reduced in mice receiving test compounds as compared to the PBS (negative control group) by the second week post treatment (Table 1). While all five derivatives exhibited antileishmanial activity in BALB/c mice, reducing both the lesion size and parasite load, C5 (100 mg/kg) significantly reduced the lesion size (0.47 ± 0.02) (Table 1) and the parasite load to 1.45 × 10^6^ compared to the PBS-treated mice (3.9 × 10^6^), as shown in Figure 2 and Figure 3.

### 3.3. Histopathology

#### 3.3.1. Effect of Test Compounds (THTT Derivatives) on Histology of the Liver

**Figure 4:**
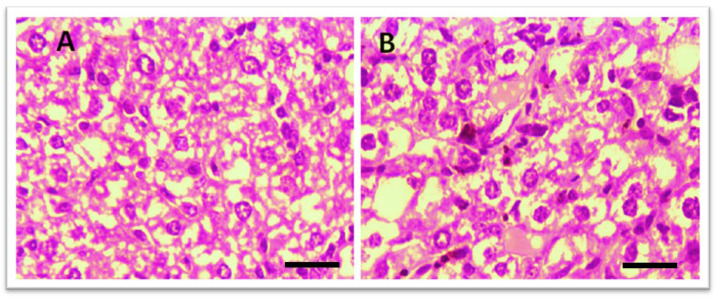
Microscopic illustration of Liver Stained with Hematoxylin and Eosin (H&E). (**A**) Normal mice; (**B**) Thiadiazine thione-derivatives treated mice with 40 × magnification (Scale bar = 10 µm).

Histological examination of the liver was performed to assess potential toxicity. Figure 4 shows that in the control group, no abnormal histopathological changes were observed. However, liver sections from THTT derivatives-treated mice did not show any major histological alterations, indicating no significant hepatic toxicity. The treatment groups showed normal liver architecture, confirming the safety of the compounds.

#### 3.3.2. Hepatic Toxicity Evaluation by Enzymatic Markers

**Figure 5:**
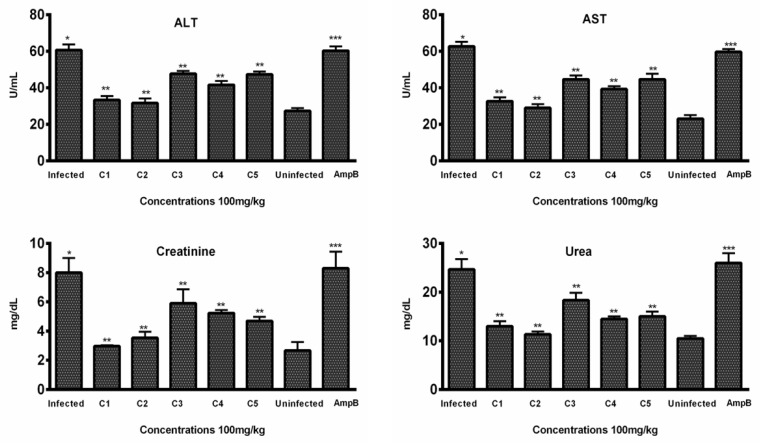
In vivo toxicity: Levels of ALT, AST, creatinine, and urea were evaluated in the sera samples of treated, infected, and untreated animals (*n* = 6). Bars indicate the mean plus standard deviation of the groups. (*), (**), and (***) indicate statistically significant difference in relation to the non-treated control. ALT (*p* < 0.01, *p* < 0.001, and *p* < 0.005); AST (*p* < 0.001, *p* < 0.005, and *p* < 0.001); creatinine (*p* < 0.01, *p* < 0.001, and *p* < 0.005); urea (*p* < 0.005, *p* < 0.01, and *p* < 0.01).

In vivo hepatic toxicity was further confirmed by evaluating the effect of test compounds on two enzymatic markers (AST and ALT) in the blood samples of the treated groups. According to the results, all compounds decreased the enzymatic level in a dose-dependent manner. Among the five compounds, C2 significantly lowered the level of ALT (31.66 ± 2.51) at 100 mg/kg body weight of mice, comparable to the level of ALT in normal mice (27.33 ± 1.527). In comparison, the Amp B treatment group exhibited significantly higher ALT levels (60.33 ± 2.30), indicating potential hepatic toxicity associated with the standard drug (Figure 5).

Similarly, C2 was also found to be effective in lowering the level of AST (29.00 ± 3.0), comparable to the level (23.0 ± 2) of normal mice, while the AST level (59.66 ± 1.52) of mice treated with Amp B was significantly higher (Figure 5), indicating the toxicity associated with the standard drug.

#### 3.3.3. Renal Toxicity Evaluation by Blood Urea and Creatinine

**Figure 6:**
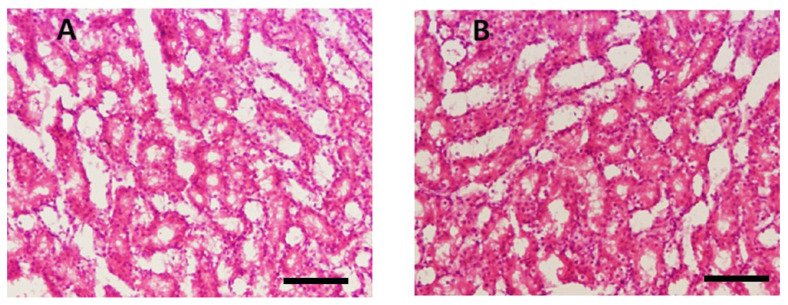
Microscopic illustration of kidney stained with hematoxylin and eosin (H&E). (**A**) Normal mice; (**B**) thiadiazine thione derivatives-treated mice with 40 × magnification (Scale bar = 10 µm).

Histopathological examination of kidney tissues showed no significant damage or toxicity in the mice treated with THTT derivatives, with no major changes in tissue architecture, such as red coloration and hemorrhagic streaks (Figure 6). Urea and creatinine levels in the blood samples were significantly lowered by C1, C2, and C5. The levels of creatinine were reduced to 33.33 ± 2.08, 31.66 ± 2.51, and 41.66 ± 2.08, respectively, in comparison to 60.33 ± 2.30 in the Amp B-treated group (Figure 5). Additionally, C2, C3, and C4 significantly reduced urea levels to 11.33 ± 0.57, 13.00 ± 1.0, and 14.50 ± 0.50, respectively, compared to the normal mice group (10.50 ± 0.50), while in the Amp B-treated group, the urea level was 26.00 ± 3.67, as seen in Figure 5. These results suggest that the THTT derivatives do not induce renal toxicity.

### 3.4. In Vivo Anti-Inflammatory Assay

To investigate the anti-inflammatory potential of the THTT derivatives, we used the carrageenan-induced paw edema model in BALB/c mice. The results demonstrated that C1 (100 mg/kg) showed the most significant activity by reducing inflammation (53.36 ± 1.00%) at the first hour and increasing it to 63.66 ± 2.08% at the fifth hour (Table 2). C5 and C4 (100 mg/kg) also exhibited notable effects, with 54.08 ± 4.12 and 56 ± 2.08 percent reductions in paw edema, respectively. All tested compounds demonstrated dose-dependent efficacy, reducing paw edema over time.

### 3.5. In Vivo Antipyretic Assay

The antipyretic potential of the THTT derivatives was evaluated in a Brewer’s yeast-induced pyrexia model. All compounds exhibited significant results (*p* < 0.05) in reducing rectal temperature by up to 1 °C over a 5 h period. C1, C2, C3, C4, and C5 (100 mg/kg) reduced the temperature within the first hour post treatment and maintained the normal temperature until the fifth hour, as shown in Table 3. The results suggest that all of the five THTT derivatives can act as potential antipyretic drugs.

### 3.6. In Vivo Analgesic Activity

**Figure 7:**
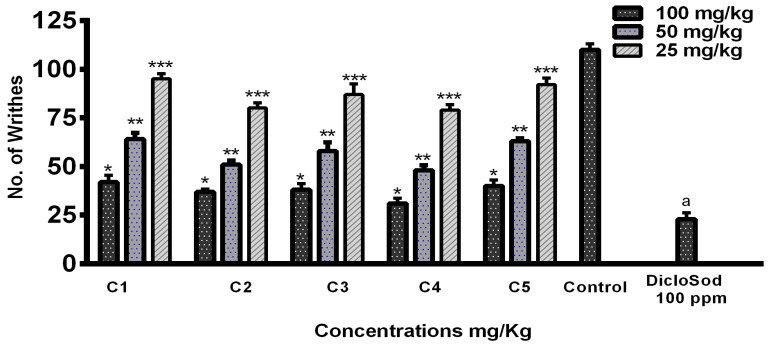
Graphical Representation of Acetic Acid-Induced Writhing Test Results. Bars Indicate the Mean plus Standard Deviation of the Groups (*n* = 6). (*), (**), (***), and (a) Indicate Statistically Significant Difference in Relation to the Non-treated Control (*p* < 0.01, *p* < 0.001, *p* < 0.005, and *p* < 0.05), Respectively.

For the antinociceptive/analgesic study of the THTT derivatives, we used acetic acid-induced abdominal writhing test, a standard model of pain. Acetic acid (1%)-induced abdominal writhing was significantly decreased by C4, C2, C3, C5, and C1 in a dose-dependent manner at 100 mg/kg. The number of writhes decreased to 35, 40, 46, 47, and 48, respectively, for these compounds compared to 117 writhes in the control group (Figure 7). The results indicate that all THTT derivatives showed significant analgesic effects, with C5 and C4 exhibiting the highest reduction in writhing behavior.

### 3.7. In Vivo Antisedative Assay

The antisedative activity of THTT derivatives was assessed at various concentrations, as mentioned below in Table 4. According to the results, all derivatives showed an antisedative effect in a dose-dependent manner; however C1 and C5 demonstrated significant effects. The number of lines crossed by mice were 94 ± 2 for C1 and 92 ± 1 for C5 (25 mg/kg group), significantly higher than the control group (131 ± 1) and the group with the standard drug Diazepam (13 ± 1). The activity/alertness declined as the concentration was increased (100 mg/kg group) significantly for C5 as compared to C1 and other derivatives.

## 4. Discussion

Thiadiazine thione derivatives were reported to possess various bioactivities, including antiproliferative [40], antibacterial [41], antiepileptic [42], antifungal [43], antileishmanial [43], antimalarial [44], antitubercular [45], trypanocidal [46], antioxidant [47], and herbicidal [48] properties. Due to the importance of the thiadiazine thione nucleus in biomedical research, this study was designed to investigate the in vivo efficacy of five selected THTT derivatives (Figure 1) for their leishmanicidal potential and to uncover the additional pharmacological benefits, such as anti-inflammatory, antinociceptive, antipyretic, and antisedative potential, at selected doses.

In the current study, two mono- and three tris-THTT derivatives were analyzed for their antileishmanial activity against *Leishmania tropica* in an in vivo experiment. The results revealed that tris-THTT derivatives (C3, C4, and C5) are more potent compared to the mono-THTT derivatives (C1 and C2). Among the tris-THTT derivatives’ in vivo treatment of infected mice, C5 significantly reduced the parasite load in the blood sample. C1 and C2 were also found to be active against *Leishmania major* promastigotes in a previous in vitro study [23]. However, in vitro testing against amastigotes was beyond the scope of this study, representing a limitation that warrants future investigation. To the best of our knowledge, this study represents the first investigation of tris-THTT derivatives, introducing a novel series of compounds. From the current study, it was observed that the introduction of three rings in the same molecule potentiated the antiprotozoal activity of the tris-THTT derivatives. Furthermore, the presence of mesityl group could be the reason for the enhanced antileishmanial activity as compared to C3 and C4. Overall, the promising antiprotozoal activity of THTT derivatives observed in this study can be attributed to the interaction of the cysteine proteinase of the parasite with isothiocyanates [49]. When the thiadiazine ring is hydrolyzed in a biological system, it yields isothiocyanates, which potentially leads to the antiparasitic activity of the derivatives [50]. The structure–activity relationship also revealed that there is key role of substituents at positions N-3 and N-5 in leishmanicidal activity. The presence of a hydrophilic group at the N-3 position or an alkyl/aryl at the N-5 position can enhance the activity [22]. Hence, the presence of a butyl group at the N-3 position makes C2 more active as compared to C1. Previous studies also reported the synthesis and leishmanicidal effect of THTT derivatives [22,51]. The present study only investigated the antileishmanial activity against one species, *L. tropica,* which is a limitation of the study. Testing against other species, such as *L. major*, could provide more comprehensive understanding of the compounds’ efficacy.

The toxicity evaluation through histopathology and by means of hepatic and kidney markers indicated that these derivatives were non-toxic and exhibited a favorable safety profile. The levels of ALT, AST, creatinine, and urea in infected mice upon administration of THTT derivatives were as low as those observed in non-infected and non-treated control mice. Histopathological examination of liver and kidney tissues did not show any significant alterations in the treated groups, suggesting that these compounds are safe for further development. The oral toxicity of THTT derivatives was previously evaluated in one study, which found moderate toxicity via oral administration but low toxicity via dermal and inhalation routes [52]. Furthermore, no ulcerogenic effects were observed in gastric mucosal cells, confirming the safety of THTT derivatives in gastric mucosal cells [53].

Inflammation is a local response to tissue damage, and basically, it is a defense mechanism of the body that is associated with granuloma formation, leukocyte infiltration, and edema. Sometimes, during inflammation, different complex mediators and events can aggravate the response [54]. Anti-inflammatory drugs such as NSAIDs are most extensively used as antipyretic and analgesic medicines. However, these drugs are associated with long-term GIT abnormalities and other adverse effects [55]. The main mechanism of NSAIDs is the inhibition of cyclooxygenase enzymes, which is responsible for prostaglandin synthesis [56]. In our study, the anti-inflammatory activity of the THTT derivatives was evaluated using the carrageenan-induced paw edema model. All the derivatives showed promising anti-inflammatory potential, with C1 (100 mg/kg) exhibiting the most significant activity by reducing inflammation (63.66%) at the fifth hour. This effect can be attributed to the presence of a lipophilic substituent in C1, particularly the n-propyl group, which was found to enhance the compound’s anti-inflammatory activity. The structure–activity relationship exposed the importance of lipophilic substituents, especially the n-propyl group, for inhibition of inflammation [57]. These findings are consistent with previous findings that also showed the importance of lipophilic substituents in enhancing anti-inflammatory activity [58]. Furthermore, the anti-inflammatory activity of the THTT nucleus was also confirmed in another study on animal models [53,59]. Our findings revealed that the anti-inflammatory potential of selected THTT derivatives could be due to inhibiting the synthesis of cyclooxygenase enzyme, just as in NSAIDs [60]. The possible improvement of the anti-inflammatory properties of these basic structures, through modulation of the ring substituents and/or further functionalization, warrants further investigation. The anti-inflammatory activity was assessed using a single model (e.g., carrageenan-induced paw edema). Testing in additional models, such as lipopolysaccharide-induced inflammation, could help confirm the findings.

The antipyretic activity of the THTT derivatives is a novel finding, as this is the first report to describe the antipyretic effects of these compounds. Previous studies on similar heterocyclic compounds, such as thiadiazole derivatives, have also shown significant antipyretic effects [38]. Our study contributes to this growing body of research by demonstrating the ability of all (C1, C2, C3, C4, and C5) derivatives to reduce body temperature in yeast-induced pyrexia in mice, maintaining normal temperature up to the fifth hour. This highlights the potential of these THTT derivatives as antipyretic agents.

The acetic acid-induced writhing test, a model commonly used to evaluate peripherally acting antinociceptive compounds, showed that all tested THTT derivatives significantly reduced abdominal writhing in mice, indicating their analgesic activity. Acetic acid induces inflammatory pain by increasing prostaglandin levels and capillary permeability [61]. Our results suggest that THTT derivatives attenuate both inflammatory and non-inflammatory pain, similar to the effects of NSAIDs. These findings are in agreement with previous reports on THTT derivatives in animal models [57,62]. Our findings are also in accordance with the previous study on Thiadiazine thione derivatives in animal models [53].

Recent studies have also highlighted the pharmacological potential of nitrogen-containing heterocyclic compounds. In this context, our investigation into the antisedative properties of selected THTT derivatives paves the way for their future therapeutic applications. Diazepam, a positive control, is widely used as an anticonvulsant, but it is associated with a low therapeutic index and several adverse effects, including headache, dizziness, and drowsiness [63]. THTT derivatives, particularly C5, exhibited significant antisedative effects at lower concentrations, and the activity of C1 was attributed to the presence of a lipophilic group. These findings support the idea that lipophilic aryl rings and hydrogen-bonding domains, as described by Dimmock’s parameter, enhance sedative activity [64]. Thiadiazine thione derivatives obey the postulates of Dimmock’s parameter, which is the prerequisite for any compound to be anticonvulsant [65]. This is in line with previous studies, which identified the structural features essential for enhancing antisedative activity [65,66].

This study provides in vivo evidence for the efficacy of THTT derivatives in treating cutaneous leishmaniasis, complementing existing in vitro studies. The research suggests that these compounds may modulate the immune response, thus relieving the inflammation associated with leishmaniasis. These findings also suggest that THTT derivatives may offer improved treatment outcomes for cutaneous leishmaniasis, particularly in regions where current treatments are ineffective or toxic. Furthermore, the pharmacodynamics properties of the THTT derivatives need to be investigated, as this is essential for understanding their in vivo behavior and clinical applications. However, the current in vivo study contributes significantly by paving the way for future studies and potential clinical applications.

## 5. Conclusions

In the present work, THTT derivatives were shown to be effective in the treatment of *Leishmania tropica* infection in mice, with a low-toxicity profile at the tested concentrations. The in vivo pharmacological efficacy was also evaluated; C5 (tris-THTT derivative) demonstrated promising leishmanicidal potential as compared to the mono-THTT derivatives. However, all of the compounds exhibited anti-inflammatory potential. The THTT derivatives demonstrated a favorable safety profile, as evidenced by the absence of significant histopathological changes in liver and kidney tissues as well as normal biochemical marker levels. These findings highlight the potential of THTT derivatives as novel therapeutic agents for cutaneous leishmaniasis treatment, with additional benefits in anti-inflammatory, analgesic, antipyretic, and antisedative effects.

Further mechanistic studies are suggested to explore the full therapeutic potential of these compounds.

## Figures and Tables

**Table 1:** Effect of various concentrations of THTT derivatives on the size of lesions (cm) in BALB/c mice infected with *L. tropica* for 15 days.

Group	Dose	Baseline Mean ± SD	Sig. *	Post-Rx Mean ± SD	Sig. **
Control	--	1.36 ± 0.04	--	2.06 ± 0.02	--
AMP	100 mg/kg	1.42 ± 0.03 ***	0.006	0.44 ± 0.02 ***	<0.001
C1	100 mg/kg	1.31 ± 0.02	0.256	0.93 ± 0.02	0.237
C2	100 mg/kg	1.34 ± 0.02	0.992	0.73 ± 0.02 ***	0.005
C3	100 mg/kg	1.40 ± 0.02	0.166	0.84 ± 0.01 ***	0.062
C4	100 mg/kg	1.29 ± 0.01 ***	0.006	0.63 ± 0.02 ***	0.001
C5	100 mg/kg	1.27 ± 0.02 ***	<0.001	0.47 ± 0.02 ***	<0.001
C1	25 mg/kg	1.31 ± 0.02	0.166	1.04 ± 0.02	0.555
C2	25 mg/kg	1.32 ± 0.02	0.375	0.87 ± 0.02	0.131
C3	25 mg/kg	1.43 ± 0.02 ***	0.001	1.22 ± 0.16	0.805
C4	25 mg/kg	1.24 ± 0.02 ***	<0.001	0.84 ± 0.01 ***	0.07
C5	25 mg/kg	1.24 ± 0.02 ***	<0.001	0.77 ± 0.01 ***	0.023
C1	50 mg/kg	1.32 ± 0.02	0.668	0.94 ± 0.02	0.296
C2	50 mg/kg	1.33 ± 0.02	0.805	0.76 ± 0.01 ***	0.023
C3	50 mg/kg	1.44 ± 0.01 ***	<0.001	1.02 ± 0.02	0.518
C4	50 mg/kg	1.29 ± 0.02 ***	0.003	0.73 ± 0.03 ***	0.005
C5	50 mg/kg	1.25 ± 0.02 ***	<0.001	0.61 ± 0.01 ***	0.001

AMP: Amphotericin B; SD: standard deviation. * Bonferroni-adjusted *p*-values of *t*-test. ** Bonferroni-adjusted *p*-values of independent-sample Kruskal–Wallis test. *** indicates significant difference when compared with control group. This one-way ANOVA compares the antileishmanial effect of Amphotericin B and test compounds at various concentrations with the control group, i.e., normal saline.

**Table 2:** The Anti-inflammatory Effect of THTT Derivatives in the Carrageenan-Induced Paw Edema in BALB/c Mice. Each Value Represents the Mean Value of Rectal Temperature ± SEM (n = 6).

Groups	1 h (Mean ± SEM)	2 h (Mean ± SEM)	3 h (Mean ± SEM)	4 h (Mean ± SEM)	5 h (Mean ± SEM)
Control	8.23 ± 2.01	8.98 ± 1.67	10.07 ± 3.44	11.34 ± 1.47	10.89 ± 1.67
Aspirin	47.02 ± 1.67 *	49.66 ± 4.02 *	53.09 ± 3.98 *	55.47 ± 2.05 *	56.67 ± 3.43 *
C1-25	14.76 ± 2.30 *	19.46 ± 2.67 *	23.41 ± 2.44 *	28.23 ± 3.78 *	29.21 ± 2.09 *
C1-50	26.56 ± 3.09 *	30.47 ± 3.43 *	34.52 ± 1.98 *	39.11 ± 2.50 *	40.52 ± 3.86 *
C1-100	53.36 ± 1.00 *	53.00 ± 4.17 *	60.66 ± 2.08 *	62.30 ± 1.52 *	63.66 ± 2.08 *
C2-25	10.78 ± 2.03 *	13.76 ± 2.56 *	17.92 ± 1.67 *	26.19 ± 3.67 *	33.11 ± 1.56 *
C2-50	23.31 ± 1.89 *	24.98 ± 2.89 *	29.47 ± 2.23 *	35.71 ± 1.98 *	41.54 ± 1.09 *
C2-100	31.67 ± 2.52 *	35.66 ± 3.51 *	40.66 ± 2.08 *	45.66 ± 2.51 *	52.33 ± 1.52 *
C3-25	22.45 ± 3.34 *	23.67 ± 3.78 *	26.11 ± 2.67 *	34.45 ± 1.47 *	38.13 ± 2.67 *
C3-50	31.67 ± 2.46 *	30.16 ± 4.67 *	37.67 ± 3.67 *	43.65 ± 2.08 *	49.21 ± 4.12 *
C3-100	40.67 ± 4.09 *	41.66 ± 4.51 *	48.00 ± 3.00 *	52.00 ± 2.01 *	52.66 ± 1.15 *
C4-25	16.34 ± 3.10 *	21.30 ± 2.56 *	22.67 ± 1.59 *	30.02 ± 4.02 *	37.23 ± 2.98 *
C4-50	28.56 ± 2.56 *	32.12 ± 4.22 *	36.11 ± 2.06 *	38.60 ± 3.67 *	48.31 ± 4.11 *
C4-100	36.66 ± 2.61 *	40.33 ± 3.51 *	45.66 ± 2.51 *	49.00 ± 3.09 *	56.00 ± 2.08 *
C5-25	19.43 ± 1.89 *	25.05 ± 3.78 *	27.41 ± 1.56 *	32.08 ± 2.49 *	42.31 ± 2.65 *
C5-50	30.67 ± 2.32 *	34.90 ± 4.10 *	38.39 ± 2.67 *	43.11 ± 1.67 *	52.33 ± 2.53 *
C5-100	40.33 ± 2.51 *	43.00 ± 3.67 *	48.33 ± 0.57 *	52.33 ± 2.51 *	54.08 ± 4.12 *

* indicates *p* value < 0.05. SEM: Standard error of mean. This one-way ANOVA analysis compares the anti-inflammatory effect of five compounds in various concentrations with control.

**Table 3:** Showing the Effect of THTT Derivatives in Yeast-induced Fever in Mice. Each Value Represents the Mean Value of Rectal Temperature.

Treatment	Dose	Normal (A) Mean ± SD	24 h (B) Mean ± SD	1 h (°C) Mean ± SD	2 h (°C) Mean ± SD	3 h (°C) Mean ± SD	4 h (°C) Mean ± SD	5 h (°C) Mean ± SD
Normal Saline	10 mL/kg	36.67 ± 0.41	39.00 ± 0.19	38.00 ± 0.18	38.67 ± 0.37	39.33 ± 0.27	38.33 ± 0.14	38.00 ± 0.23
Aspirin	100 mg/kg	36.67 ±0.32	38.67 ± 0.49	37.67 ± 0.39	37.33 ± 0.23 *	37.67 ± 0.56 *	37.67 ± 0.17 *	37.67 ± 0.14
C1	25 mg/kg	36.33 ± 0.15	39.33 ± 0.23	38.67 ± 0.26	38.67 ± 0.41	38.67 ± 0.27 *	38.67 ± 0.24	38.67 ± 0.40 *
C1	50 mg/kg	36.00 ± 0.51 *	39.00 ± 0.25	38.67 ± 0.29	38.33 ± 0.38	38.00 ± 0.37 *	38.33 ± 0.24	38.00 ± 0.00
C1	100 mg/kg	36.00 ± 0.22 *	38.67 ± 0.37	37.67 ± 0.17	37.67 ± 0.14 *	38.67 ± 0.43 *	37.67 ± 0.21 *	37.23 ± 0.19 *
C2	25 mg/kg	36.45 ± 0.23	38.33 ± 0.44 *	38.00 ± 0.31	38.33 ± 0.45	38.33 ± 0.44 *	39.00 ± 0.41 *	38.67 ± 0.14 *
C2	50 mg/kg	36.67 ± 0.34	39.33 ± 0.27	38.67 ± 0.21	38.67 ± 0.21	38.67 ± 0.17 *	38.67 ± 0.14	38.67 ± 0.24 *
C2	100 mg/kg	36.30 ± 0.28	38.67 ± 0.49	37.67 ± 0.30	37.33 ± 0.36 *	37.67 ± 0.56 *	37.67 ± 0.16 *	37.67 ± 0.04
C3	25 mg/kg	36.00 ± 0.33 *	38.75 ± 0.18	38.67 ± 0.20	38.33 ± 0.40	38.00 ± 0.57 *	38.33 ± 0.14	38.00 ± 0.00
C3	50 mg/kg	36.00 ± 0.19 *	38.09 ± 0.22 *	37.67 ± 0.48	37.67 ± 0.51 *	38.67 ± 0.27 *	38.01 ± 0.47	37.81 ± 0.19
C3	100 mg/kg	36.67 ± 0.26	38.33 ± 0.10 *	38.00 ± 0.17	38.33 ± 0.40	38.33 ± 0.14 *	38.00 ± 0.19	36.67 ± 0.34 *
C4	25 mg/kg	36.67 ± 0.49	38.67 ± 0.25	37.67 ± 0.31	37.33 ± 0.11 *	37.67 ± 0.16 *	37.67 ± 0.17 *	37.67 ± 0.47
C4	50 mg/kg	36.35 ± 0.20	39.33 ± 0.29	38.67 ± 0.18	38.67 ± 0.41	38.67 ± 0.37 *	38.67 ± 0.24	38.67 ± 0.44 *
C4	100 mg/kg	36.15 ± 0.52 *	39.00 ± 0.17	38.67 ± 0.13	38.33 ± 0.32	38.00 ± 0.27 *	37.33 ± 0.14 *	37.00 ± 0.00 *
C5	25 mg/kg	36.04 ± 0.24 *	38.67 ± 0.11	38.33 ± 0.18	37.67 ± 0.38	38.67 ± 0.24 *	38.67 ± 0.09	37.67 ± 0.29
C5	50 mg/kg	36.67 ± 0.33	38.33 ± 0.43 *	38.00 ± 0.15	38.33 ± 0.17	38.33 ± 0.14 *	37.00 ± 0.26 *	37.75 ± 0.19
C5	100 mg/kg	36.33 ± 0.48	38.23 ± 0.44 *	37.67 ± 0.27	38.33 ± 0.19 *	38.33 ± 0.24 *	37.33 ± 0.14 *	37.33 ± 0.34 *

* indicates *p* value < 0.05. SD: Standard deviation. A: mean of normal body temperature; B: temperature after carrageenan treatment. This one-way ANOVA analysis compares the antipyretic activity of normal saline (control) treatment with aspirin and experiment compounds in various concentrations.

**Table 4:** Antisedative Effect of THTT Derivatives on Mice Model (*n = 6*) at Varying Concentrations. Each Value Represents the Mean Value and ± SD.

Groups	Line Crossed	Sig. *
	Mean ± SD	
Control	131 ± 2	Ref.
Diazepam	13 ± 1 **	<0.001
C1-100	50 ± 1 **	0.007
C1-50	74 ± 2 **	0.05
C1-25	94 ± 2	0.773
C2-100	55 ± 1 **	0.013
C2-50	78 ± 2	0.097
C2-25	81 ± 2	0.222
C3-100	65 ± 1 **	0.026
C3-50	81 ± 2	0.278
C3-25	86 ± 1	0.458
C4-100	34 ± 2 **	0.001
C4-50	47 ± 2 **	0.003
C4-25	79 ± 2	0.138
C5-100	20 ± 1 **	<0.001
C5-50	34 ± 1 **	0.001
C5-25	92 ± 1	0.65

* Bonferroni-adjusted *p*-values of independent-sample Kruskal–Wallis test. ** indicates significant difference when compared with control group.

## Data Availability

All data presented in the current study are included in this article.
